# Multifunctional Hierarchical Surface Structures by Femtosecond Laser Processing

**DOI:** 10.3390/ma11050789

**Published:** 2018-05-12

**Authors:** Clemens Kunz, Frank A. Müller, Stephan Gräf

**Affiliations:** Otto Schott Institute of Materials Research (OSIM), Friedrich Schiller University Jena, Löbdergraben 32, 07743 Jena, Germany; clemens.kunz@uni-jena.de (C.K.); stephan.graef@uni-jena.de (S.G.)

**Keywords:** fs-laser, hierarchical surface structures, fused silica, silanization, optical properties, wettability, negative replica casting, polystyrene

## Abstract

Hierarchical surface structures were fabricated on fused silica by using a fs-laser with a pulse duration *τ* = 300 fs and a wavelength *λ* = 512 nm. The resulting surface structures were characterized by scanning electron microscopy, atomic force microscopy and white light interference microscopy. The optical properties were analyzed by transmittance measurements using an integrating sphere and the wettability was evaluated by measuring the water contact angle *θ*. The silanization of structured fused silica surfaces with trichloro(1H,1H,2H,2H-perfluorooctyl)silane allows to switch the wettability from superhydrophilic (*θ* = 0°) to superhydrophobic behavior with *θ* exceeding 150°. It was shown that the structured silica surfaces are a suitable master for negative replica casting and that the hierarchical structures can be transferred to polystyrene. The transmittance of structured fused silica surfaces decreases only slightly when compared to unstructured surfaces, which results in high transparency of the structured samples. Our findings facilitate the fabrication of transparent glass samples with tailored wettability. This might be of particular interest for applications in the fields of optics, microfluidics, and biomaterials.

## 1. Introduction

Multifunctional surfaces consisting of hierarchical micro- and nanostructures are provided and optimized by nature for a wide range of requirements [1]. Among others, they include the realization of superhydrophobic self-cleaning properties known from the lotus leaf [2], anti-reflective optical properties provided by the wings of the *Greta oto.* butterfly [3], and the fog-harvesting ability of the Namib dessert beetle, which allows the insect to survive in hostile environments [4,5]. Biological systems stimulated intense research aiming on the transfer of nature’s functional principles to technical applications. For this purpose, different techniques like replica casting of natural surfaces [6], nanolithography [7,8,9], chemical etching [10], deposition of nanoparticles on surfaces [11,12], and ultra-short pulsed laser processing [1,13,14] have been investigated.

The utilization of ultra-short pulsed lasers (fs-lasers) for the fabrication of biomimetic surfaces has gained rapidly increasing attention in recent decades [15,16]. Based on the very short pulse duration and the large laser peak fluence, this method allows to structure almost all classes of materials with the desired high precision and without the occurrence of noticeable heat affected zones [17]. Beyond, fs-lasers allow the generation of so-called laser-induced periodic surface structures (LIPSS). Since their first observation by Birnbaum in 1965 [18], LIPSS were intensively investigated in order to unveil their formation process as well as the dependence of influencing parameters [1,15,16,17,19,20,21,22,23,24,25,26]. There are two types of LIPSS, the high-spatial frequency LIPSS (HSFL) and the low-spatial frequency LIPSS (LSFL) [21,27]. The first ones that arise below the ablation threshold, have a period much smaller than the incident laser wavelength and generally are aligned perpendicular to the electrical field vector in the case of dielectrics [16,21,28]. The latter ones that are created near or above the ablation threshold have periods near the utilized laser wavelength and are mainly aligned parallel to the electrical field vector. Due to the dependence on laser parameters, tailor-made orientations and periods in the range of a few nanometers up to 1 µm are adjustable. Thus, surface nanostructuring with LIPSS enables engineering of material surfaces concerning wettability [29], optical properties [30], and tribology [31].

In terms of wettability, different approaches were presented on the basis of hierarchical surface structures to obtain superhydrophobic surfaces [12,32,33]. However, to the best of our knowledge, only very few studies investigated the control of wettability by utilizing a fs-laser to process hierarchical structures on glass. Superhydrophobic hierarchical surface structures with linear alignment exhibiting enhanced wear resistance after fs-laser treatment and silanization were created by Boinovich et al. [34]. Furthermore, Stroj et al. [35] should be mentioned, who structured quartz surfaces and achieved superhydrophobicity after silanization with –CF_2_. Mimicking the fog harvesting ability of the Namib dessert beetle, Kostal et al. [5] used a fs-laser to structure borosilicate glass samples. After a subsequent silanization step, the hierarchical surface structure exhibited adjustable wettability.

In the present study, we investigated the fabrication of hierarchical surface structures on fused silica by fs-laser processing with the objective to control the wetting behavior while simultaneously preserving the high transparency of the glass samples. Such multifunctional surfaces might be of particular interest for applications in the fields of optics, photovoltaics, and microfluidics [1,36,37]. Beyond, the suitability of hierarchically structured glass surfaces to be used as master in the negative replica casting process of polymers is demonstrated. This process allows the production of multifunctional surfaces on flexible substrates and opens new possibilities to produce e.g., superhydrophobic biomaterials.

## 2. Materials and Methods

Hierarchical surface structures were fabricated on fused silica (GVB, Herzogenrath, Germany) using the second harmonic (*λ* = 512 nm) of a diode pumped Yb:KYW thin disc fs-laser system (JenLas D2.fs, Jenoptik, Jena, Germany) in air under ambient conditions. The emitted linearly polarized laser radiation was characterized by a pulse duration *τ* = 300 fs and a repetition frequency *f*_rep_ = 100 kHz. The ablation threshold and the focal spot diameter of the Gaussian beam was determined to be 2*w*_f_ = (13 ± 0.5) µm using the method proposed by Liu [38] by using *N* = 5 laser pulses and a repetition frequency of *f*_rep_ = 100 kHz. Furthermore, by taking the same parameters the degree of disorder was evaluated over single spot experiments with different laser fluences *F* from 4.5 J/cm^2^ up to 18.2 J/cm^2^ and *N* = 5. For hierarchical structuring, the fused silica surface was scanned with a focussed laser beam using a galvanometer scanner (IntelliScan14, Scanlab, Puchheim, Germany) and a 100 mm f-Theta objective (JENar, Jenoptik, Jena, Germany). The scanning velocity was set to *v* = 1 m/s and a lattice like scan mode with a hatch distance of *d* = 15 µm in vertical and horizontal direction was set. This scan mode was repeated *S* = 50, which results in an effective pulse number per spot of *N* = (*π* × *w*_f_^2^ × *f*_rep_ × 2*S*)/(*v* × *d*) = 88. The pulse energy *E*_imp_ was chosen to 12 µJ resulting in a laser peak fluence *F* = (2 × *E*_imp_)/(*π* × *w*_f_^2^) = 18.2 J/cm^2^. Before and after the laser processing, the fused silica samples were ultrasonically cleaned in acetone and isopropanol.

The wettability was modified by silanization of the fused silica surface using trichloro(1H,1H,2H,2H-perfluorooctyl)silane (Alfa Aesar, Karlsruhe, Germany). For this purpose, the sample was placed in a desiccator close to a 100 µl drop of silane. Using a vacuum pump for 30 min, the silane was deposited on the fused silica surface by gas phase condensation. After deposition, the samples were stored in a furnace (LM-312.27, Linn High Therm, Hirschbach, Germany) at a temperature of *T_CVD_* = 100 °C for 5 h. Replica casting of the surface was performed with polystyrene (Carl Roth, Karlsruhe, Germany) using a stamp with a load of 5 kg at *T_Cast_* = 250 °C. The wettability of the sample surfaces with distilled water was analyzed by contact angle measurements (drop-shape analyzer 10 Mk2, Krüss, Hamburg, Germany). For this purpose, a droplet was measured in the sessile drop mode with a minimum droplet volume of 2.4 µL.

The optical properties of the samples were evaluated using a xenon-lamp (Tunable PowerArc Illuminator, Optical Building Blocks, Birmingham, New Jersey, USA) and an integrating sphere (IS236A-4, Thorlabs, Newton, NJ, USA). With a detector (Maya2000 Pro, Ocean Optics, Largo, FL, USA), the wavelength ranges from 400 to 1100 nm were investigated. The accumulation of the measurement was set to 1000 and the integration time was 10 ms.

The morphology of the samples was characterized by scanning electron microscopy (SEM) (Sigma VP, Carl-Zeiss, Oberkochen, Germany) at an accelerating voltage of 5 kV using the secondary electron detector and by white light interference microscopy (WLIM) (CCI HD, Taylor Hobson, Weiterstadt, Germany) equipped with a 50× objective. From the WLIM data the roughness ratio *r* between the real surface and the projected surface was calculated as well as the ratio *ϕ*_s_ of wetted surface to projected surface. Moreover, the topography of the sample surfaces was analyzed by atomic force microscopy (AFM) (NanoWizard 4, JPK Instruments, Berlin, Germany) working in the QI contact mode with a cantilever spring constant 0.14 N/m and a nominal tip radius of 2 nm.

## 3. Results and discussion

### 3.1. Surface Morphology

**Figure 1:**
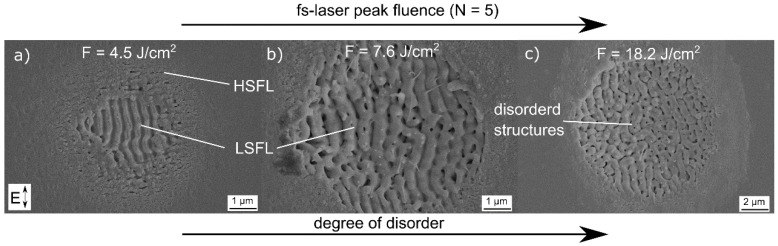
SEM micrographs of fused silica surfaces upon irradiation with *N* = 5 successive laser pulses using a laser peak fluence (**a**) *F* = 4.5 J/cm^2^; (**b**) *F* = 7.6 J/cm^2^; and (**c**) *F* = 18.2 J/cm^2^. Note the different scale bar in (**c**).

Figure 1 shows SEM micrographs of fused silica surfaces upon the irradiation with *N* = 5 successive laser pulses using a fs-laser peak fluence *F* = 4.5 J/cm^2^ (Figure 1a), *F* = 7.6 J/cm^2^ (Figure 1b), and *F* = 18.2 J/cm^2^ (Figure 1c). At the lowest value *F* = 4.5 J/cm^2^, the micrograph reveals the formation of periodic structures with different spatial periods. They can be attributed to high-spatial frequency LIPSS (HSFL) and low-spatial frequency LIPSS (LSFL), respectively [21,27].

The formation of both types of LIPSS depends on the spatial distribution of the laser fluence in the Gaussian focal spot [25]. Using the method proposed by Liu [38], the calculated threshold for the formation of LSFL is 3.6 J/cm^2^ (*λ* = 512 nm, *f*_rep_ = 100 kHz, *τ* = 300 fs) for fused silica, which is significantly smaller than the ablation threshold of 5.1 J/cm^2^ for fused silica at a laser wavelength *λ* = 1025 nm determined by Gräf et al. [28]. This change could be explained by the different laser wavelength. Fused silica has a band gap *E*_g_ of 9 eV [39]. For the utilized laser wavelength of 512 nm (*E*_ph_ ≈ 2.8 eV) the probability of multi-photon absorption is higher than for a longer wavelength with lower photon energy. Thus, the ablation threshold decreases [40]. It can be observed that, in the intense center of the spot, LSFL are formed with an alignment parallel to the electrical field vector *E*. The corresponding spatial period was measured to 350 nm by fast Fourier transform analysis of the SEM micrograph. Thus, the spatial period is slightly smaller than the initial laser wavelength *λ*. This is typical for LSFL on fused silica and was also reported for other glasses [22,28]. The HSFL are generated in the less-intense outer area of the focal spot. Here, the spatial periods are much smaller than *λ* and the alignment is perpendicular to *E*. Increasing *F* to 7.6 J/cm^2^ (Figure 1b) leads to an increase of the area covered with LSFL, HSFL are only barely visible. A further increase of *F* to 18.2 J/cm^2^ leads to the formation of structures that are not exclusively aligned strictly parallel to *E*. These less ordered structures are characterized by spatial periods around 580 nm, i.e., larger than *λ*. A similar behavior on glasses after fs-laser irradiation with *λ* = 400 nm and *τ* = 150 fs was reported by Seifert et al. [41]. They attributed the formation of laser-induced disordered structures in the bottom of laser ablation craters to self-organization processes. Considering the relatively large value of *F*, the increased disorder and the larger periods might also be explained by melt formation and hydrodynamic effects driven by surface tension [42].

**Figure 2:**
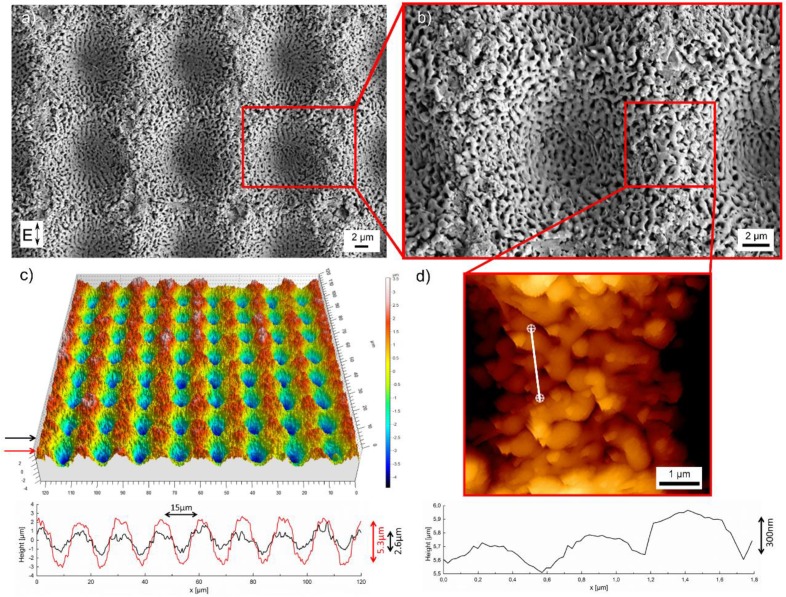
SEM micrographs of the fs-laser-induced hierarchical surface structures on fused silica: (**a**) overview and (**b)** detailed view; (**c**) three-dimensional image of the microstructure and corresponding height profiles obtained from WLIM (black and red arrows correspond to the same colors in the profiles); (**d**) AFM micrograph of the nanostructure and height profile measured along the white line.

In order to create hierarchical surface structures on fused silica, we used the largest value of the fs-laser peak fluence *F* = 18.2 J/cm^2^ and scanned the surface at a velocity of 1 m/s and a hatch distance of 15 µm. As illustrated in Figure 2a, the surface obtained in this way exhibits a characteristic periodic microstructure that results from the specific scan pattern. Moreover, disordered nanostructures can be observed, that superimpose the microstructure (Figure 2b). A three-dimensional micrograph of the resulting hierarchical structure recorded with a WLIM is shown in Figure 2c.

The micrographs reveal that the surface topography is characterized by a periodicity of 15 µm, which corresponds to the lattice like laser scanning with a hatch distance of 15 µm in vertical and horizontal direction. Information about the surface height is given by the height profiles in Figure 2c along the red and black arrow, respectively. It is shown that different profiles of the surface height were realized. The respective maximum depths are 5.3 µm along the red arrow and 2.6 µm long the black arrow. The difference could be explained by the Gaussian intensity distribution of the fs-laser beam. Considering a spot size of 2*w*_f_ = (13 ± 0.5) µm and a hatch distance of 15 µm, the depth of 5.3 µm refers to the scanning line were the maximum intensity of the Gaussian laser beam hits the surface. Consequently, the depth of 2.6 µm results from the overlap of the scanning lines and the lower intensity of the Gaussian laser beam. The AFM micrograph in Figure 2d shows the topography of the superimposed nanostructure. A height profile along the white line was taken to determine its spatial period and height. The height profile reveals that the measured size of around 650 nm approximates the values measured during the single spot experiments (Figure 1c). The height of the structures was determined to be around 300 nm, which is a typical value for laser-induced surface structure on glasses [28].

### 3.2. Negative Replica Casting

**Figure 3:**
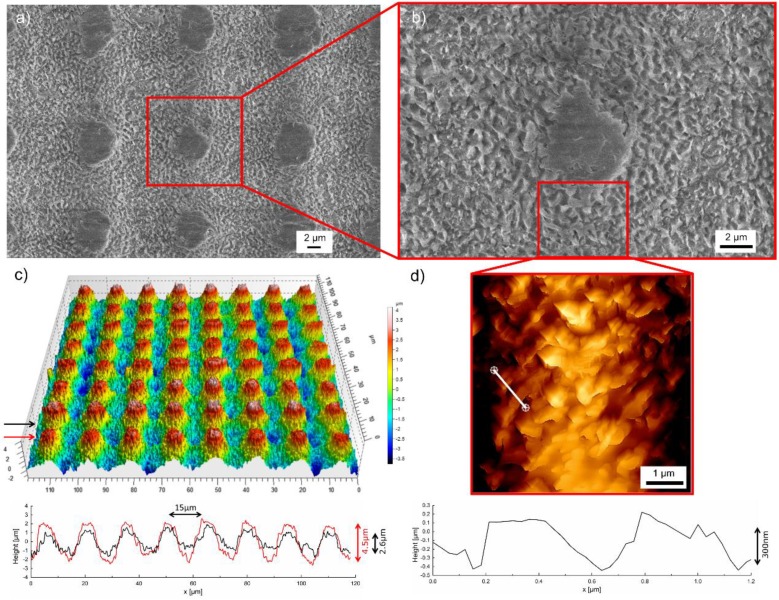
SEM micrographs of a polystyrene surface after negative replica casting of the fs-laser-induced hierarchical surface structures on fused silica (**a**) overview and (**b**) detailed view; (**c**) three-dimensional image of the microstructure and corresponding height profiles obtained from WLIM (black and red arrows correspond to the same colors in the profiles); (**d**) AFM micrograph of the nanostructure and corresponding height profile measured along the white line.

The previously produced hierarchical silica surface structures were used as a master for the negative replica casting of polystyrene (PS), because replicas made of PS already demonstrated their suitability to achieve specific surface properties, e.g., wettability or biocompatibility [43,44,45]. The negative replica surfaces (Figure 3) were characterized analogously to the laser structured fused silica sample.

SEM micrographs of cast PS surfaces (Figure 3a,b) confirm that the hierarchical micro- and nanostructures are transferred successfully to the polymer surface. A three-dimensional image of the microstructure with the corresponding height profiles obtained from WLIM is shown in Figure 3c. Similar to the hierarchical structures on the fused silica master, the periodical micropattern shows the same periodicity of about 15 µm. The height profile taken in the direction of the black arrow (Figure 3c) reveals the same profile height of 2.6 µm as measured for fused silica. However, the height profile taken in the direction of the red arrow demonstrates a maximum height of 4.5 µm, which is about 0.8 µm smaller than on the fused silica master. Consequently, a casting of the entire structural depth using polystyrene was not achieved. This is also indicated by the smooth surface areas - i.e., the flattened hills—in the SEM micrographs (Figure 3a,b). The nanostructure of the replica surface and the respective height profile were evaluated by AFM measurements (Figure 3d). It is shown that the disordered nanostructure fabricated on fused silica can be adequately transferred to polystyrene. The height and the spatial period were determined to be around 300 nm and 500 nm, respectively. Thus, both structural sizes are in the same order as measured for the fused silica master. The slightly reduced special period results from the shrinkage of the polymer during cooling from 250 °C to room temperature. The topography of the laser structured fused silica surfaces remained intact even with repeated replica casting and therefore allows multiple usage. These findings demonstrate the ability to transfer the specific hierarchical surface structures fabricated by fs-laser processing on fused silica to polystyrene, which allows engineering polymer surfaces with this kind of tailored surface structures. In this context it has to be noted that the direct fabrication of similarly sized hierarchical structures on polymers using a fs-laser is not that simple due to differing material properties (e.g., absorption behavior, formation process, melting behavior, and chemical modification). For a deeper insight into the LIPSS formation on polymers the interested reader is referred to references [46,47,48,49,50]. However, to the best of our knowledge, a direct laser approach that combines microstructuring and LIPSS is still missing. Structures produced by e.g., direct laser interference pattering (DLIP) are one magnitude larger than LIPSS and a tailored disorder could not be achieved [51].

### 3.3. Wettability

**Figure 4:**
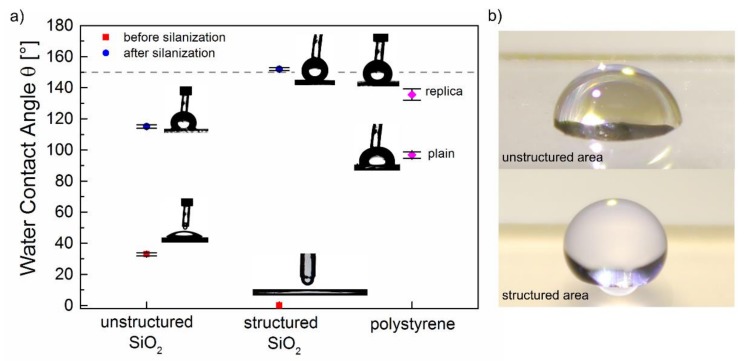
Analysis of the wettability of fused silica and polystyrene: (**a**) water contact angle *θ* of unstructured and structured fused silica before and after silanization in comparison to the contact angle of both surfaces that have been replicated by using polystyrene; (**b**) photographs of a water droplet illustrating the wettability of the unstructured and structured fused silica surface after silanization.

The analysis of the water contact angle *θ* of all investigated samples is illustrated in Figure 4. Unstructured fused silica surfaces are characterized by a relatively small contact angle *θ* = 32.9° ± 1.5° indicating hydrophilic behavior. After laser processing, the structured silica surfaces exhibit superhydrophilic behavior with *θ* = 0°. After silanization of these surfaces with trichloro(1H,1H,2H,2H-perfluorooctyl)silane, the wettability switched to a superhydrophobic with *θ* = 152° ± 0.9° and *θ* = 115° ± 1.1° for structured and unstructured silica surfaces, respectively (Figure 4b). In the case of the replica structures, the contact angle of plain polystyrene surfaces was determined to *θ* = 96.7° ± 2.0° which is in good agreement with literature [45]. After negative replica casting of the hierarchically structured glass surfaces, the resulting polystyrene structures became more hydrophobic, which is indicated by an increase of *θ* to 135.6° ± 3.6°.

The results reveal that the wettability of silica surfaces can be tailored in the full range of superhydrophilic and superhydrophobic behavior by adjusting their surface roughness and chemistry. This can be explained by applying models proposed by Wenzel and by Cassie and Baxter [52,53]. According to Wenzel, the contact angel *θ*^W^ of a rough substrate surface is given by cos*θ*^W^ = *r* × cos*θ* with *θ* being the contact angle of the flat surface and *r* being a roughness factor that represents the ratio of the real surface area to its horizontal projection. Consequently, the increase of *r* leads to an increasing contact angle for a hydrophobic surface and to a decreasing contact angle for a hydrophilic surface [52]. For the unstructured fused silica with *θ* = 32.9° ± 1.5°, the increased surface roughness caused by the laser-induced hierarchical structures leads to *θ* = 0°. To explain this drastic decrease by using Wenzel’s law, a theoretical value of at least *r* = 1.19 would be required. However, the roughness factor of the corresponding surface (Figure 2) measured by WLIM was even higher with *r* = 1.26, resulting in the complete spreading of the water droplet on the entire surface. The contact angle of silanized fused silica increased from *θ* = 115° ± 1.1° for unstructured samples to *θ* = 152° ± 0.9° for hierarchically structured samples. Generally, wetting states with *θ* exceeding 150° can only be explained by utilizing the model of Cassie and Baxter, which considers the presence of air bubbles under the droplet on a rough surface. Following this model, only small areas of the surface (*ϕ*_s_) are in direct contact to the liquid and the Cassie–Baxter contact angle *θ*^CB^ is given by cos*θ*^CB^ = *ϕ*_s_ × cos*θ* + *ϕ*_s_ − 1 [53]. Taking into account the contact area *ϕ*_s_ = 0.13 calculated by WLIM, a theoretical contact angle *θ*^CB^ = 157° can be calculated, which is in good agreement with the experimentally determined contact angle. This also confirms that the applied condensation process leads to the formation of silane monolayers which only slightly reduce the roughness of the original silica surface. On PS surfaces, the increased surface roughness caused by replica casting leads an increase of *θ* from 96.7° ± 0.9° to 135.6 ± 3.6°. With the roughness ratio *r* = 1.33 determined by WLIM, Wenzel’s law is not suitable to predict this drastic increase of *θ*. In the case of the replica surface, the calculated contact area is *ϕ*_s_ = 0.19. By using the equation of Cassie–Baxter a *θ*^CB^ = 146° can be predicted, which is close to our measured value. Thus, the observed behavior might be explained by an intermediate state between Wenzel and Cassie–Baxter [54,55].

### 3.4. Optical Properties

**Figure 5:**
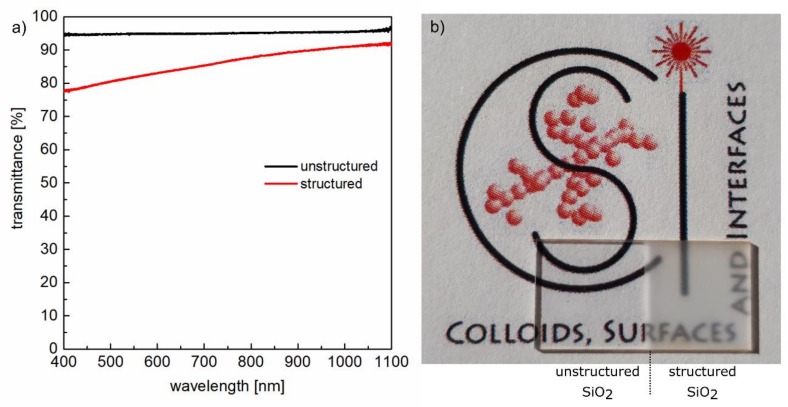
Optical characterization of the unstructured and structured fused silica surface: (**a**) transmittance measured in the wavelength range 400 to 1100 nm using an integrating sphere and (**b**) visualization of the optical effect of the surface structures by means of a photography of the sample transparency.

Figure 5a shows the influence of the hierarchical surface structures on the transmittance of the samples measured with an integrating sphere in the wavelength range between 400 and 1100 nm. It becomes evident that the transmittance of the unstructured sample remains almost constant at about 95% in the entire investigated spectral range. The remaining part to 100% transmission is attributable to the reflection at both interfaces of the bulk material. For a single surface of a non-absorbing medium, this specular reflectance can be calculated at normal incidence using Fresnel’s formulas according to *R* = ((*n* − 1)/(*n* + 1))^2^ with *n* being the refractive index of the medium. In the case of fused silica, *n* decreases only marginal from *n* = 1.4701 at *λ* = 400 nm to *n* = 1.4492 at *λ* = 1100 nm [56], which predicts an almost constant value of *R* ≈ 3.5%. Considering both interfaces, a reflectance *R* ≈ 7% is to be expected. Deviations to the experimentally obtained value of *R* ≈ 5% can be explained by the fact that Fresnel’s formulas refer to an ideal flat surface. The spectral dependency of the transmittance of the structured sample shows a decrease in the investigated wavelength range starting from *T* ≈ 90% at *λ* = 1100 nm to *T* ≈ 78% at *λ* = 400 nm. This behavior results from an increased surface roughness that causes diffuse reflectance at the sample surface. The latter increases with decreasing size of the surface structures and therefore grows in importance when the wavelength of the measurement radiation comes in the range of the spatial period of the laser-induced nanostructures [57,58]. However, despite the hierarchical surface structures, the initial high transparency of the fused silica samples was almost completely retained, which is also illustrated by the photograph in Figure 5b. Both surfaces, the unstructured and the laser structured surface show the visibility of an underlying text. These findings demonstrate the possibility to create tailored superhydrophilic and superhydrophobic surfaces without affecting the sample transparency, which might be of potential interest for optical applications, smart windows and facades as well as for microfluidic devices and sensors [36,59].

## 4. Conclusions

We studied the fabrication of hierarchical surface structures on fused silica. It was shown that the superimposed micro- and nanostructures lead to a reduction of the water contact angle, i.e., to superhydrophilic behavior. After silanization of the structured surfaces, the wettability switched to superhydrophobic behavior with contact angles exceeding 150°. Optical characterization confirmed a high transparency of the structured glass samples. The transfer of these structures to polystyrene demonstrated the suitability of structured fused silica masters for negative replica casting. Thus, our findings facilitate the fabrication of multifunctional, transparent surfaces with tailored wettability and might be of particular interest for applications in the fields of microfluidics, optics, and biomaterials.
